# Supplementary material for: TP53 mutations emerge with HDM2 inhibitor SAR405838 treatment in de-differentiated liposarcoma
Source: Nat Commun. 2016 Aug 31;7:12609. doi: 10.1038/ncomms12609 (PMC5013668; doi:10.1038/ncomms12609)
Supplement: Supplementary Information — Supplementary Figures 1-4 and Supplementary Tables 1-2. [file ncomms12609-s1.pdf]

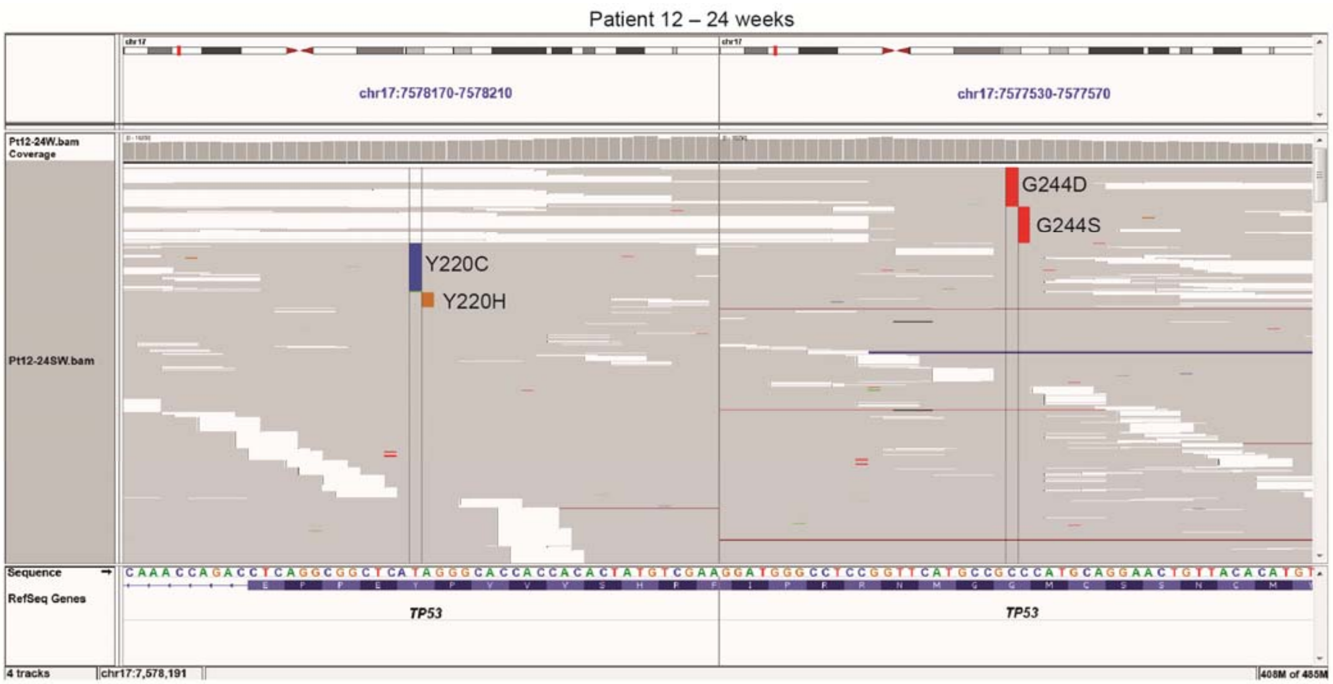


**Supplementary Figure 1: IGV view of multiple variants within the 24-week cfDNA sample from patient 12**

Blue, brown and red colors indicate sequencing reads containing a *TP53* variant within the sequencing alignment. Variants affecting neighboring bases are not on the same DNA strand, suggesting that these variants originated from separate clones in the tumor.
IGV, integrative genomics viewer.

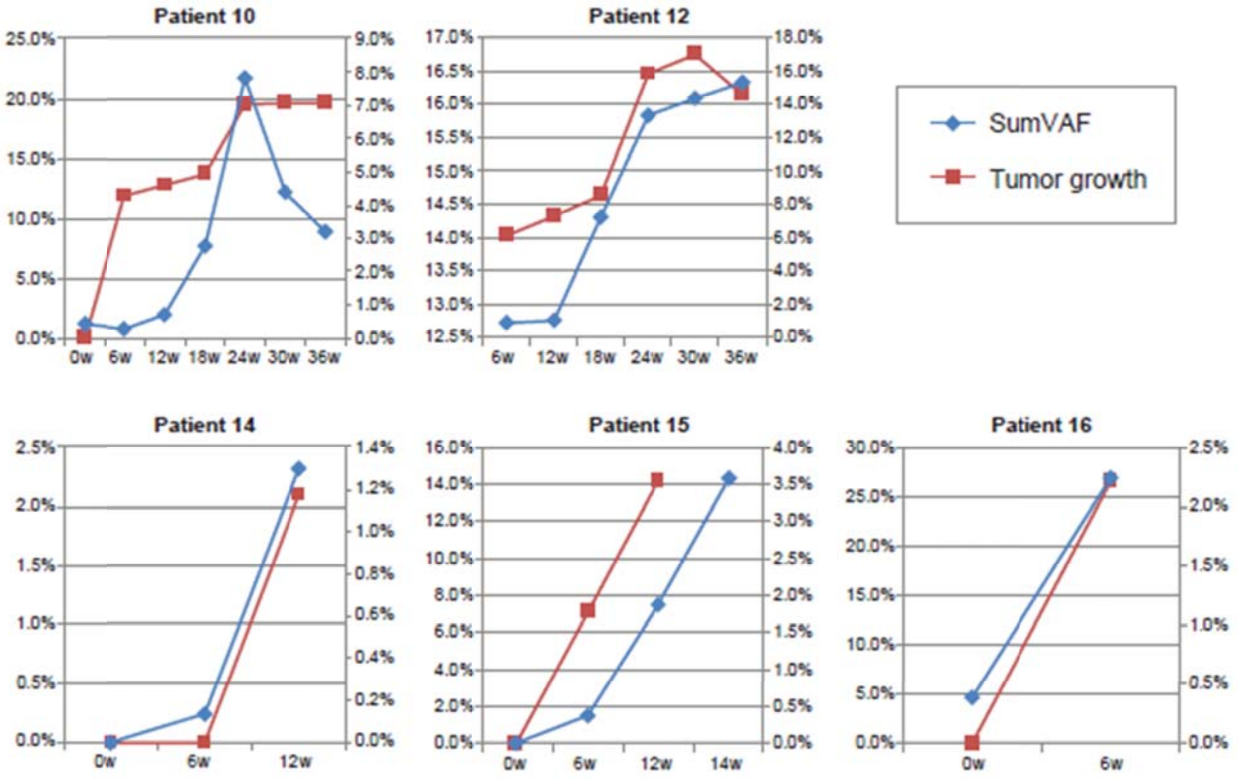


**Supplementary Figure 2: Comparison of tumor growth and total *TP53* mutation burden in cfDNA**

Tumor growth is presented as percentage change in tumor size by computed tomography measurement relative to baseline measurement (Y axis 1, left). Total *TP53* mutation burden is sum of all *TP53* variant allelic fractions (VAF; Y axis 2, right). X axis represents time on treatment (weeks).

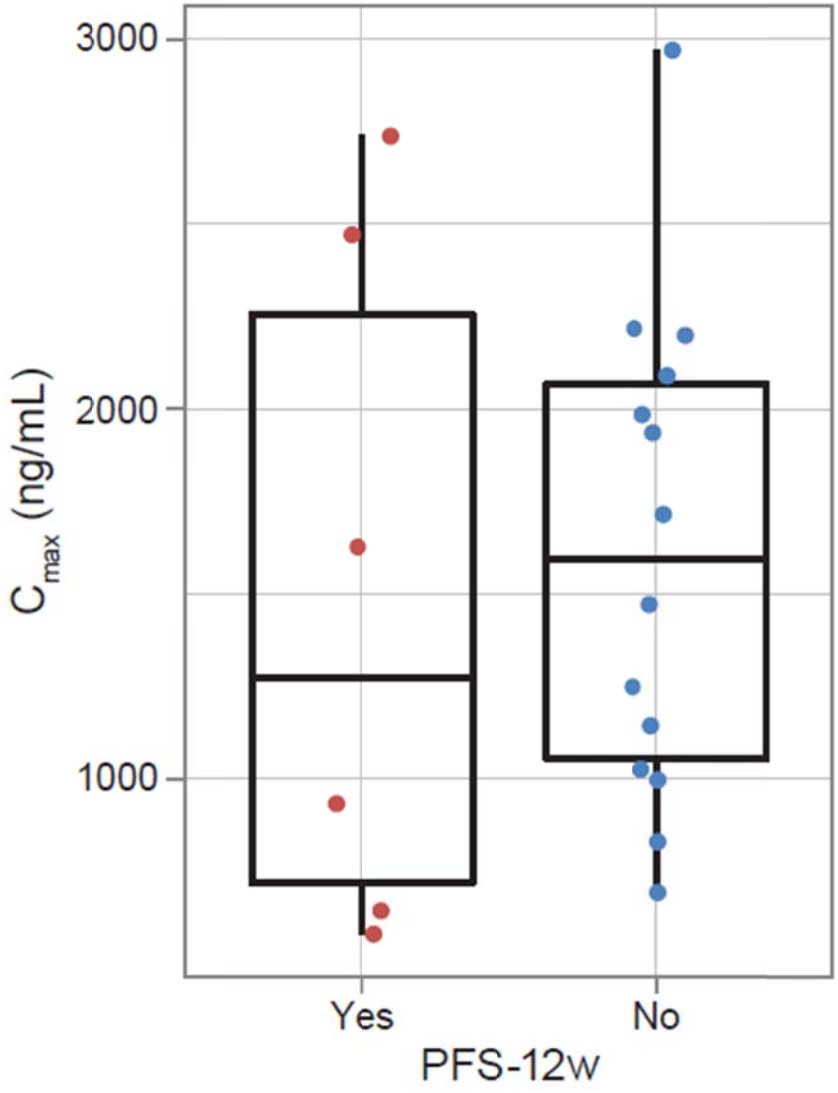


**Supplementary Figure 3: Comparison of $C_{max}$ at baseline (Cycle 1, Day 1) for patients with and without progression-free survival at 12 weeks (PFS-12w)**

Box plots with actual data points are shown (n = 20). Center line indicates median value. Bottom and top of the whiskers represent the smallest and largest non-outlier values respectively. Lower and top end of the box indicate the 1st and 3rd quartile respectively.

**Supplementary Figure 4: cfDNA yields in patients with DDLPS receiving SAR2405838**

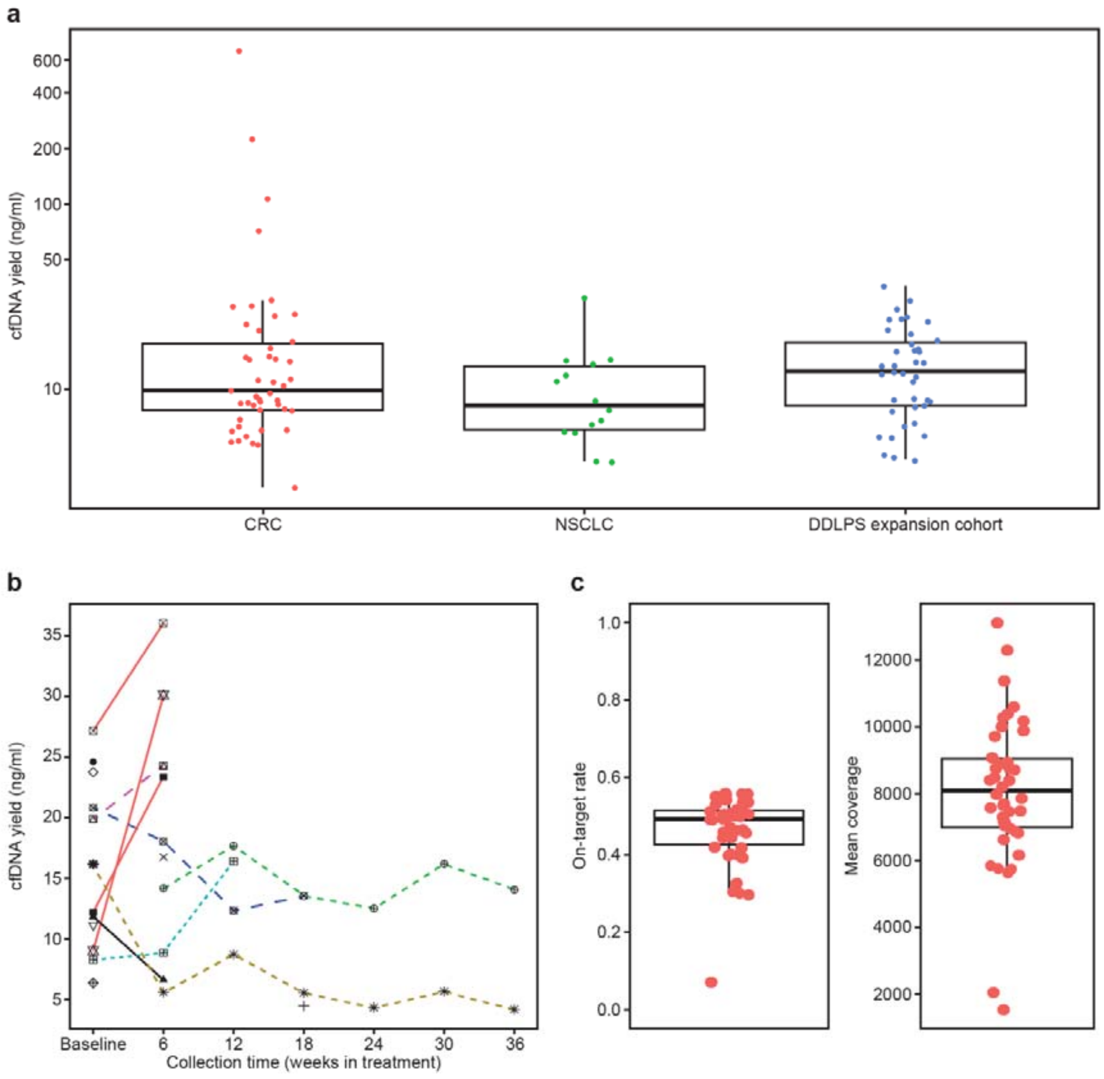


(a) Plasma cfDNA was isolated from patients participating in the DDLPS expansion cohort. The cfDNA yield was similar to that observed in the 60 matched CRC and NSCLC tumor and plasma pairs. Box plots with actual data points are shown. CRC, colorectal cancer (n = 46); DDLPS, de-differentiated liposarcoma (n = 39); NSCLC, non-small cell lung cancer (n = 14). (b) The plasma cfDNA yield from patients participating in the DDLPS expansion cohort did not consistently increase or decrease over time. (c) cfDNA sequencing QC results from the DDLPS expansion cohort suggest that most libraries met our targets of 40% on-target rate and 8000X mean sequence coverage. Box plots with actual data points are shown (n = 39).

cfDNA, cell-free DNA; CRC, colorectal cancer; DDLPS, de-differentiated liposarcoma; NSCLC, non-small cell lung cancer.

**Supplementary Table 1: Summary of prior treatments, progression-free survival at 12 weeks (PFS-12w), tumor volume at baseline and tumor volume changes during treatment**

| | | | | **Tumor volume change (%)** | | | | | | | | | | |
|---|---|---|---|---|---|---|---|---|---|---|---|---|---|---|
| **Patient ID** | **Prior treatments** | **PFS-12w** | **Tumor vol at baseline (cm$^3$)** | base line | 6w | 12w | 18w | 24w | 30w | 36w | 42w | 48w | (grey) | Plasma available for this study |
| 3 | Adriamycin, ifosfamide, antineoplastic agents | No | 10.15 | 0 | 28.94737 | | | | | | | | (orange) | *TP53* VAF ≥ 1.0% |
| 4 | Doxorubicine, Endoxan | No | 289.6 | 0 | 14.92537 | | | | | | | | | |
| 5 | | Yes | 310.7 | 0 | -8.374384 | -15.27094 | -19.21182 | -23.64532 | -24.13793 | -23.64532 | -24 | -24 | | |
| 6 | Doxorubicin, trabectedin | Yes | 27.3 | 0 | -0.9771987 | 4.560261 | 1.954397 | 11.07492 | | | | | | |
| 7 | Doxorubicin | No | 64.4 | 0 | 22.73099 | | | | | | | | | |
| 8 | Cytoxan, adriamycin, vincristine, ifosfamide, etoposide, mesna, gemcitabine, taxotere, protein kinase inhibitors | No | 385.8 | 0 | 11.45308 | 30.02018 | | | | | | | | |
| 9 | Doxorubicine, gemcitabine, docetaxel, trabectedin, DTIC, Gemzar, Taxotere, protein kinase inhibitors | No | 27.3 | 0 | 15.53218 | 33.04456 | | | | | | | | |
| 10 | Gemcitabine, docetaxel, doxorubicin, ifosfamide | Yes | 224.97 | 0 | 11.85468 | 12.81071 | 13.67113 | 19.50287 | 19.59847 | 19.59847 | | | | |
| 11 | | No | 347 | 0 | 23.57195 | | | | | | | | | |
| 12 | Docetaxel, gemcitabine, sorafenib, antineoplastic agents. | Yes | 124.8 | 0 | 14.02439 | 14.32927 | 14.63415 | 16.46342 | 16.76829 | 16.15854 | | | | |
| 13 | Doxorubicin, cyclophosphamide | No | 2281.5 | 0 | 5.252525 | | | | | | | | | |
| 14 | | Yes | 191 | 0 | 0 | 2.094241 | | | | | | | | |
| 15 | Dexamethasone, gemcitabine, docetaxel, antineoplastic agents, palbociclib | Yes | 1006.3 | 0 | 7.094594 | 14.18919 | 33.10811 | | | | | | | |
| 16 | gemcitabine, palbociclib, alisertib, vismodegib, antineoplastic agents | No | 1795 | 0 | 26.6171 | | | | | | | | | |
| 17 | Palbociclib, gemcitabine | No | 606 | 0 | 22.76451 | | | | | | | | | |
| 18 | | No | 80.6 | 0 | 23.73737 | | | | | | | | | |
| 19 | Palbociclib | No | 91.2 | 0 | 16.54135 | | | | | | | | | |
| 20 | Evofosfamide, doxorubicin, gemcitabine, navelbine | No | | 0 | | | | | | | | | | |

## Supplementary Table 2: *TP53* probe sequences

| | |
|---|---|
| Hs_TP53_e1_1 | /5Biosg/TGTCCAGCTTTGTGCCAGGAGCCTCGCAGGGGTTGATGGGATTGGGGTTTTCCCCTCCCATGTGCTCAAGACTGGCGCTAAAAGTTTTGAGCTTCTCAAAAGTCTAGAGCCACCGTCCAG |
| Hs_TP53_e1_2 | /5Biosg/GGAGCAGGTAGCTGCTGGGCTCCGGGGACACTTTGCGTTCGGGCTGGGAGCGTGCTTTCCACGACGGTGACACGCTTCCCTGGATTGGGTAAGCTCCTGACTGAACTTGATGAGTCCTCT |
| Hs_TP53_e2_1 | /5Biosg/GGCCCAGGTGACCCAGGGTTGGAAGTGTCTCATGCTGGATCCCCACTTTTCCTCTTGCAGCAGCCAGACTGCCTTCCGGGTCACTGCCATGGAGGAGCCGCAGTCAGATCCTAGCGTCGA |
| Hs_TP53_e2_2 | /5Biosg/GCCCCCTCTGAGTCAGGAAACATTTTCAGACCTATGGAAACTGTGAGTGGATCCATTGGAAGGGCAGGCCCACCACCCCCACCCCAACCCCAGCCCCCTAGCAGAGACCTGTGGGAAGCG |
| Hs_TP53_e2_3 | /5Biosg/AAAATTCCATGGGACTGACTTTCTGCTCTTGTCTTTCAGACTTCCTGAAAACAACGTTCTGGTAAGGACAAGGGTTGGGCTGGGGACCTGGAGGGCTGGGGACCTGGAGGGCTGGGGGGC |
| Hs_TP53_e3_1 | /5Biosg/GAGGACCTGGTCCTCTGACTGCTCTTTTCACCCATCTACAGTCCCCCTTGCCGTCCCAAGCAATGGATGATTTGATGCTGTCCCCGGACGATATTGAACAATGGTTCACTGAAGACCCAG |
| Hs_TP53_e3_2 | /5Biosg/GTCCAGATGAAGCTCCCAGAATGCCAGAGGCTGCTCCCCCCGTGGCCCCTGCACCAGCAGCTCCTACACCGGCGGCCCCTGCACCAGCCCCCTCCTGGCCCCTGTCATCTTCTGTCCCTT |
| Hs_TP53_e3_3 | /5Biosg/CCCAGAAAACCTACCAGGGCAGCTACGGTTTCCGTCTGGGCTTCTTGCATTCTGGGACAGCCAAGTCTGTGACTTGCACGGTCAGTTGCCCTGAGGGGCTGGCTTCCATGAGACTTCAAT |
| Hs_TP53_e4_1 | /5Biosg/GCTGAGGTGGGAAGATCACTTGAGGCCAGGAGATGGAGGCTGCAGTGAGCTGTGATCACACCACTGTGCTCCAGCCTGAGTGACAGAGCAAGACCCTATCTCAAAAAAAAAAAAAAAAA |
| Hs_TP53_e4_2 | /5Biosg/GAAAAGCTCCTGAGGTGTAGACGCCAACTCTCTCTAGCTCGCTAGTGGGTTGCAGGAGGTGCTTACGCATGTTTGTTTCTTTGCTGCCGTCTTCCAGTTGCTTTATCTGTTCACTTGTGC |
| Hs_TP53_e4_3 | /5Biosg/CCTGACTTTCAACTCTGTCTCCTTCCTCTTCCTACAGTACTCCCCTGCCCTCAACAAGATGTTTTGCCAACTGGCCAAGACCTGCCCTGTGCAGCTGTGGGTTGATTCCACACCCCCGCC |
| Hs_TP53_e4_4 | /5Biosg/CGGCACCCGCGTCCGCGCCATGGCCATCTACAAGCAGTCACAGCACATGACGGAGGTTGTGAGGCGCTGCCCCCACCATGAGCGCTGCTCAGATAGCGATGGTGAGCAGCTGGGGCTGGA |
| Hs_TP53_e5_1 | /5Biosg/TTAGGTCTGGCCCCTCCTCAGCATCTTATCCGAGTGGAAGGAAATTTGCGTGTGGAGTATTTGGATGACAGAAACACTTTTCGACATAGTGTGGTGGTGCCCTATGAGCCGCCTGAGGTC |
| Hs_TP53_e6_1 | /5Biosg/TCCTAGGTTGGCTCTGACTGTACCACCATCCACTACAACTACATGTGTAACAGTTCCTGCATGGGCGGCATGAACCGGAGGCCCATCCTCACCATCATCACACTGGAAGACTCCAGGTCA |
| Hs_TP53_e7_1 | /5Biosg/TAGGACCTGATTTCCTTACTGCCTCTTGCTTCTCTTTTCCTATCCTGAGTAGTGGTAATCTACTGGGACGGAACAGCTTTGAGGTGCGTGTTTGTGCCTGTCCTGGGAGAGACCGGCGCA |
| Hs_TP53_e7_2 | /5Biosg/CAGAGGAAGAGAATCTCCGCAAGAAAGGGGAGCCTCACCACGAGCTGCCCCCAGGGAGCACTAAGCGAGGTAAGCAAGCAGGACAAGAAGCGGTGGAGGAGACCAAGGGTGCAGTTATGC |
| Hs_TP53_e8_1 | /5Biosg/CACCTTTCCTTGCCTCTTTCCTAGCACTGCCCAACAACACCAGCTCCTCTCCCCAGCCAAAGAAGAAACCACTGGATGGAGAATATTTCACCCTTCAGGTACTAAGTCTTGGGACCTCTT |
| Hs_TP53_e9_1 | /5Biosg/TTGTAGCTAACTAACTTCAGAACACCAACTTATACCATAATATATATTTTAAAGGACCAGACCAGCTTTCAAAAAGAAAATTGTTAAAGAGAGCATGAAAATGGTTCTATGACTTTGCCT |
| Hs_TP53_e9_2 | /5Biosg/GATACAGATGCTACTTGACTTACGATGGTGTTACTTCCTGATAAACTCGTCGTAAGTTGAAAATATTGTAAGTTGAAAATGGATTTAATACACCTAATCTAAGGAACATCATAGCTTAGC |
| Hs_TP53_e10_1 | /5Biosg/GCTGCAGATCCGTGGGCGTGAGCGCTTCGAGATGTTCCGAGAGCTGAATGAGGCCTTGGAACTCAAGGATGCCCAGGCTGGGAAGGAGCCAGGGGGGAGCAGGGCTCACTCCAGGTGAGT |
| Hs_TP53_e11_1 | /5Biosg/CTTCTGTCTCCTACAGCCACCTGAAGTCCAAAAAGGGTCAGTCTACCTCCCGCCATAAAAAACTCATGTTCAAGACAGAAGGGCCTGACTCAGACTGACATTCTCCACTTCTTGTTCCCC |
| Hs_TP53_e11_2 | /5Biosg/ACTGACAGCCTCCCACCCCCATCTCTCCCTCCCCTGCCATTTTGGGTTTTGGGTCTTTGAACCCTTGCTTGCAATAGGTGTGCGTCAGAAGCACCCAGGACTTCCATTTGCTTTGTCCCG |
| Hs_TP53_e11_3 | /5Biosg/GGGCTCCACTGAACAAGTTGGCCTGCACTGGTGTTTTGTTGTGGGGAGGAGGATGGGGAGTAGGACATACCAGCTTAGATTTTAAGGTTTTTACTGTGAGGGATGTTTGGGAGATGTAAG |
| Hs_TP53_e11_4 | /5Biosg/AAATGTTCTTGCAGTTAAGGGTTAGTTTACAATCAGCCACATTCTAGGTAGGGGCCCACTTCACCGTACTAACCAGGGAAGCTGTCCCTCACTGTTGAATTTTCTCTAACTTCAAGGCCC |
| Hs_TP53_e11_5 | /5Biosg/ATATCTGTGAAATGCTGGCATTTGCACCTACCTCACAGAGTGCATTGTGAGGGTTAATGAAATAATGTACATCTGGCCTTGAAACCACCTTTTATTACATGGGGTCTAGAACTTGACCCC |
| Hs_TP53_e11_6 | /5Biosg/CTTGAGGGTGCTTGTTCCCTCTCCCTGTTGGTCGGTGGGTTGGTAGTTTCTACAGTTGGGCAGCTGGTTAGGTAGAGGGAGTTGTCAAGTCTCTGCTGGCCCAGCCAAACCCTGTCTGAC |
| Hs_TP53_e11_7 | /5Biosg/AACCTCTTGGTGAACCTTAGTACCTAAAAGGAAATCTCACCCCATCCCACACCCTGGAGGATTTCATCTCTTGTATATGATGATCTGGATCCACCAAGACTTGTTTTATGCTCAGGGTCA |
| Hs_TP53_e11_8 | /5Biosg/ATTTCTTTTTTCTTTTTTTTTTTTTTTTTTTCTTTTTCTTTGAGACTGGGTCTCGCTTTGTTGCCCAGGCTGGAGTGGAGTGGCGTGATCTTGGCTTACTGCAGCCTTTGCCTCCCCGGCT |
| Hs_TP53_e11_9 | /5Biosg/CGAGCAGTCCTGCCTCAGCCTCCGGAGTAGCTGGGACCACAGGTTCATGCCACCATGGCCAGCCAACTTTTGCATGTTTTGTAGAGATGGGGTCTCACAGTGTTGCCCAGGCTGGTCTCA |
| Hs_TP53_e11_10 | /5Biosg/AACTCCTGGGCTCAGGCGATCCACCTGTCTCAGCCTCCCAGAGTGCTGGGATTACAATTGTGAGCCACCACGTCCAGCTGGAAGGGTCAACATCTTTTACATTCTGCAAGCACATCTGCA |
| Hs_TP53_e11_11 | /5Biosg/TTTTCACCCCACCCTTCCCCTCCTTCTCCCTTTTTATATCCCATTTTTATATCGATCTCTTATTTTACAATAAAACTTTGCTGCCACCTGTGTGTCTGAGGGGTGAACGCCAGTGCAGGC |
